# Copper concentration data for water, sediments, and vegetation of urban stormwater ponds treated with copper sulfate algaecide

**DOI:** 10.1016/j.dib.2020.105982

**Published:** 2020-07-03

**Authors:** Mary G. Lusk, Kylie Chapman

**Affiliations:** Soil and Water Sciences Department, Gulf Coast Research and Education Center, University of Florida, United States

**Keywords:** Stormwater ponds, Copper, Pennywort, Dollarweed, Copper sulfate algaecides, Copper phytoremediation

## Abstract

We characterized copper (Cu) concentrations in the water, sediments, and shoreline plants of stormwater ponds in the urban Tampa, Florida area. We selected 6 urban residential stormwater ponds that receive summer wet season (May to September) Cu sulfate applications at least twice a month. We collected triplicate water and sediment samples from each pond and analyzed for Cu, as well as nutrient pools (inorganic N and P) and a suite of other physicochemical properties (e.g., water temperature, pH, conductivity, alkalinity, etc.). We analyzed shoreline plant tissue samples for Cu. The raw dataset provides values for Cu concentrations in water, sediments and plant tissue, and other measured parameters in water and sediments.

This dataset is important for characterizing the fate and potential mobility of Cu in freshwater ponds treated with Cu sulfate algaecides. This applied research data will provide baseline understanding of Cu concentrations in water, sediments, and select plant tissue samples, providing insights on potential toxicity of Cu and any threats that Cu sulfate algaecides may pose to aquatic organisms and downstream waters. This dataset can also inform future research designs aimed at elucidating the effects of Cu on denitrifying bacteria and N removal in stormwater pond ecosystems. Finally, the plant tissue data shows variable Cu concentrations among plant species, and this data can inform future phytoremediation experiments.

**Specifications Table**

**Table:** 

**Subject**	Environmental Science (General)
**Specific subject area**	Copper accumulation in urban stormwater ponds
**Type of data**	Tables Graphs
**How data were acquired**	A water quality multiparameter sonde (YSI, Inc.) was used to measure *in situ* temperature, surface dissolved oxygen, conductivity, and pH of water samples. We used an inductively coupled plasma (ICP) spectrophotometer to measure Cu concentrations in water, sediment extracts, and plant tissues. We used LaMotte's KH/Alkalinity Test Kit 4491-DR-01 to measure the total alkalinity of the water samples. We used a continuous flow analyzer to measure inorganic N and P pools in water and sediment extracts.
**Data format**	Raw Filtered Analyzed
**Parameters for data collection**	The following parameters were measured: Water—alkalinity, temperature, surface dissolved oxygen, pH, conductivity, Cu, NH_4_—N, NO_3_—N, Orthophosphate-P Sediments—exchangeable Cu, NH_4_—N, NO_3_—N, total N Plant tissues—Cu
**Description of data collection**	Triplicate water and sediment samples and plant tissue samples were collected from 6 urban stormwater ponds in August 2019. All ponds had been previously treated with copper sulfate algaecides. Water samples were collected at random points approximately 1 m from the pond shorelines. Sediment samples were collected from random locations within the ponds and at a depth of 15 cm. Plant tissue was collected from dominant plant species within 0.5 m of the pond shorelines, both within and outside the ponds. Samples were placed in HDPE bottles or plastic zip-top bags, transported back to the lab on ice and processed for analysis immediately or stored at 4 °C up to 1 week.
**Data source location**	Institution: University of Florida, Gulf Coast Research and Education Center City/Town/Region: Hillsborough County, Florida Country: United States of America Latitude and longitude (and GPS coordinates) for collected samples/data: 27.411934, −82.428501
**Data accessibility**	With the article

**Value of the data**

• The data provide information about Cu concentrations in environmental pools (water, sediment, plant tissue) of stormwater ponds, a type of waterbody that numbers in the tens of thousands in Florida alone [1] and is common in many other urban areas.
• Aquatic biologists, stormwater scientists and engineers, and researchers can benefit from the data. The data can be used by those interested in heavy metal (Cu) contamination in freshwater bodies, in phytoremediation of Cu contamination, and in how we can manage copper sulfate algaecide applications in urban waters.
• These data may be used to inform future experiments related to phytoremediation of Cu in soils and sediments.
• These data may be used to inform future experimental designs aimed at learning the effects of Cu on aquatic organisms, including the microbial community responsible for N transformations and removal, thus providing insights on the connections between algal management strategies and nutrient cycling in the ponds.

## 1 Data description

Stormwater ponds are a type of stormwater control measure used in urban environments to primarily reduce flooding associated with urban runoff, though they are often expected to perform some level of pollutant removal as well [2, 3]. They often provide only minimal removal of nutrients, including nitrogen (N) and phosphorus (P) [[4], [5], [6], [7]–8]. As N and P from urban runoff accumulate in stormwater ponds, it is not uncommon for the ponds to become highly eutrophic and support dense populations of aquatic vegetation and planktonic and filamentous algae.

Copper sulfate (CuSO_4_ • 5H_2_O) is the active ingredient in several trademarked algaecides used to control algae in stormwater ponds and other freshwater systems [9,10]. Copper sulfate applications of 1.0 to 1.5 ppm (equivalent to 0.26 to 0.39 ppm as Cu^2+^) are often applied to stormwater ponds on a weekly or biweekly basis to control rapid proliferation of algae [11]. Copper (Cu) can accumulate in the tissues of living organisms and is toxic to many aquatic organisms [12,13]. Moreover, Cu can inhibit the activity of nitrifying and denitrifying bacteria, thus decreasing the N removal capacity of stormwater ponds [14]. This dataset is the product of an exploratory analysis of Cu concentrations in water, sediments, and plant tissue from stormwater ponds in the Tampa, Florida area.

**Fig. 1:**
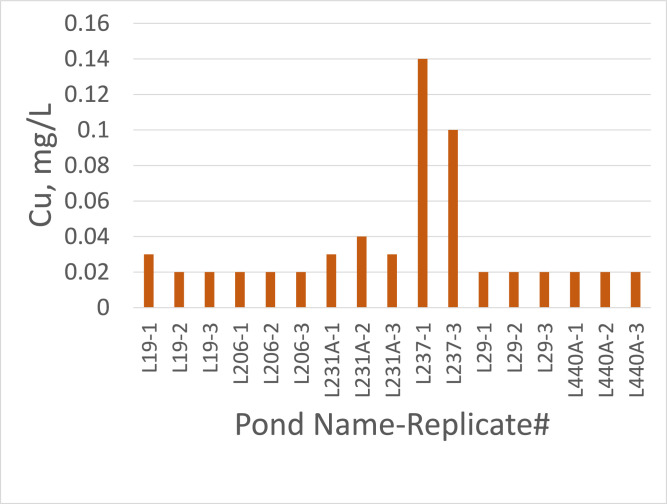
Copper concentrations in water samples from 6 urban stormwater ponds treated with copper sulfate algaecide.

**Fig. 2:**
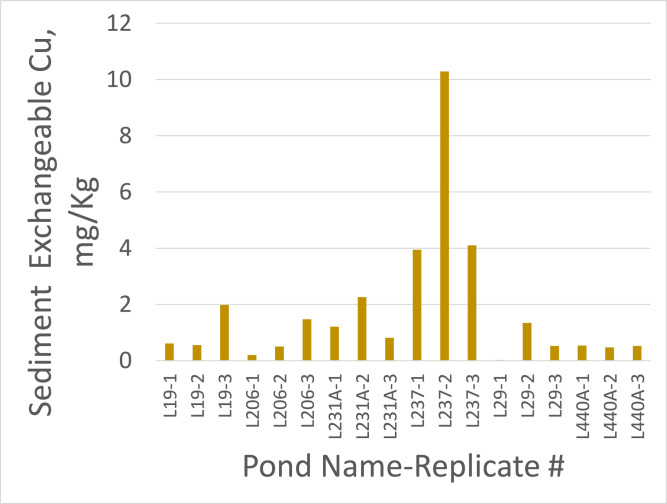
Exchangeable copper concentrations in sediment samples from 6 urban stormwater ponds treated with copper sulfate algaecide.

**Fig. 3:**
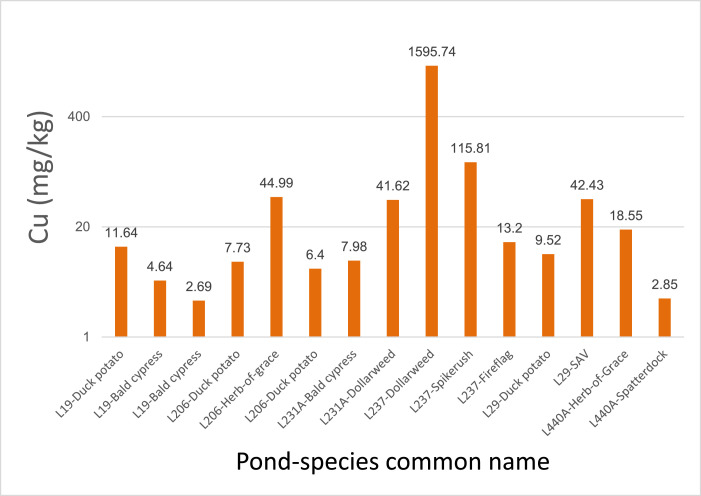
Copper concentrations in plant tissue samples from the shorelines of 6 urban stormwater ponds. Note logarithmic scale of *y*-axis. See Table 1 for plant species scientific names and a description of plant parts that were sampled.

All data values are in Supplementary File 1 in the worksheet titled “Data.” The dataset contains entries for water, sediment, and plant tissue, with reported values for Cu concentrations as well as basic physicochemical properties (temperature, DO, pH, etc.) and nutrient pools (N and P) for water and sediments. Copper concentrations were consistently higher in sediments than in water samples. With the exception of pond L237, Cu in water seldom exceeded 0.02 mg/L and exchangeable Cu in sediment was generally below 2 mg/Kg (Figs. 1 and 2). Pond L237 had water Cu concentrations as high as 0.14 mg/L and exchangeable sediment Cu concentrations as high as 10.3 mg/Kg. Plant tissue samples had variable Cu concentrations, with pennywort, also known as dollarweed (*Hydrocotyle umbellata*), accumulating up to 1596 mg/Kg Cu (Fig. 3).

A Pearson correlation matrix of water and sediment parameters is in Supplementary File 1 in the worksheet titled “Correlation Matrix.” Copper concentrations in water were positively and strongly correlated (correlation coefficient >0.50) with NH_4_—N, ortho-P, and total dissolved P concentrations in water and with sediment Cu and P concentrations. Copper concentrations in sediment were positively and strongly correlated with water pH; Cu, ortho-P and total dissolved P concentrations in water; and with the sediment P concentration.

## 2 Experimental design, materials, and methods

### 2.1 Site description

We selected six residential stormwater ponds in Lakewood Ranch, a medium-density neighborhood in Brandenton, Florida, which is part of the Tampa Bay watershed. The six ponds receive stormwater runoff from neighborhood streets, roofs, and sidewalks and are frequently impaired by excess aquatic vegetation and planktonic algae. Neighborhood demographics and stormwater parameters in the neighborhood have been characterized in previous studies [15]. The ponds receive copper sulfate applications of 1.0 to 1.5 ppm (equivalent to 0.26 to 0.39 ppm as Cu^2+^) at least twice a month during the summer rainy season (May to September).

### 2.2 Water, sediment and plant tissue sampling

Samples were collected in August 2019, the middle of the summer rainy season and during a time in which copper sulfate was being applied to ponds biweekly. We selected 3 random sampling points approximately 1.2 m from the shoreline of each of the 6 ponds. At each point, we used a YSI water quality sonde to take a baseline *in situ* measurement of water temperature, surface dissolved oxygen, pH, and conductivity. We then collected 250 ml of pond water from each of the 3 points in all ponds. Water samples were placed into acid-washed HDPE bottles and then placed in a cooler on ice for transport back to the lab. Triplicate sediment samples were collected from the top 15 cm of each sampling point, placed in plastic bags and transported to the lab in a cooler. On the day of sampling we also walked the shoreline of each pond and made an inventory of non-turfgrass vegetation growing within 0.5 m of the shoreline, both inside and outside the ponds (included submerged aquatic vegetation, if it was present, growing just inside the pond shoreline). For each plant species, we collected leaf, flower, stem, or shoot samples (Table 1). Plant samples were placed in plastic bags and transported back to the lab in a cooler.

**Table 1:** Description of plants sampled in this study.

Pond name	Plant species, common name	Plant species, scientific name	Description of sampled tissue
L19	Duck potato	*Sagittaria landifolia*	Flowers and leaves
L19	Bald cypress	*Taxodium distichum*	Twig and needles
L19	Bald cypress	*Taxodium distichum*	Twig and needles
L206	Duck potato	*Sagittaria landifolia*	Leaf
L206	Herb-of-grace	*Bacopa sp.*	Whole plant
L206	Duck potato	*Sagittaria landifolia*	Leaf
L231A	Bald cypress	*Taxodium distichum*	Twig and needles
L231A	Dollarweed (aka pennywort)	*Hydrocotyle umbellata*	Runners (stem with leaf and roots)
L237	Dollarweed (aka pennywort)	*Hydrocotyle umbellata*	Runners (stem with leaf and roots)
L237	Spikerush	*Eleocaris interstincta*	Stalk
L237	Fireflag	*Thalia geniculata*	Leaf
L29	Duck potato	*Sagittaria ladifolia*	Leaf and flower
L29	Submerged aquatic vegetation	*Elecharis baldwinii*	Whole plant
L440A	Spatterdock	*Nuphar advena*	Leaf and flower
L440A	Herb-of-grace	*Bacopa sp.*	Whole plant

### 2.2 Water, sediment, and plant tissue analysis

Water samples were analyzed in the lab for Cu^2+^, NH_4_—N, NO_3_—N, and PO_4_–P. We used EPA method 200.7 to analyze for Cu^2+^ by ICP spectrophotometry. Water samples were 0.45µ-filtered, preserved to pH <2.0 with sulfuric acid, and were analyzed within 28 days for inorganic nutrient forms (NO_3_-, NH_4_+, and PO_4_+) at the University of Florida IFAS Analytical Research Laboratory using air-segmented continuous autoflow analyzers via EPA methods 353.2, 350.1, and 365.1, respectively. Water samples were analyzed for total alkalinity using LaMotte's KH/Alkalinity Test Kit 4491-DR-01 and direct reading titrator method. Sediment samples were air dried and passed through a #10 sieve prior to extraction and analysis. For sediment exchangeable Cu 1.0 g of oven dried (105 °C for 12 h) of sediment was extracted with 25 ml of 1 M NH_4_NO_3_. Extracts were then analyzed for Cu via ICP spectrophotometry. Sediment nutrient pools (inorganic N and P) were determined after extracting 20 g of air dry sediment with 150 ml of 2 N KCl and analyzing extracts in the same manner as water samples. Plant tissue samples were rinsed with deionized water, placed on a lab bench to air dry and then ground with a plant tissue grinder. They were analyzed for Cu via ICP spectrophotometry.

### 2.3 Statistical analysis

We used Microsoft Excel to create a Pearson correlation matrix for measured water and sediment parameters.

## Declaration of Competing Interest

The authors declare that they have no known competing financial interests or personal relationships which have, or could be perceived to have, influenced the work reported in this article.

## Appendix Supplementary materials

Image, application 1

Image, application 2
